# Group 2 Innate Lymphoid Cells Are Redundant in Experimental Renal Ischemia-Reperfusion Injury

**DOI:** 10.3389/fimmu.2019.00826

**Published:** 2019-04-16

**Authors:** Guy J. M. Cameron, Kelly M. Cautivo, Svenja Loering, Simon H. Jiang, Aniruddh V. Deshpande, Paul S. Foster, Andrew N. J. McKenzie, Ari B. Molofsky, Philip M. Hansbro, Malcolm R. Starkey

**Affiliations:** ^1^Priority Research Centre's GrowUpWell and Healthy Lungs, School of Biomedical Sciences and Pharmacy, The University of Newcastle, Callaghan, NSW, Australia; ^2^Hunter Medical Research Institute, New Lambton Heights, NSW, Australia; ^3^Department of Laboratory Medicine, University of California, San Francisco, San Francisco, CA, United States; ^4^Department of Immunology and Infectious Disease, John Curtin School of Medical Research, Australia National University, Canberra, ACT, Australia; ^5^Department of Renal Medicine, The Canberra Hospital, Canberra, ACT, Australia; ^6^The John Hunter Children's Hospital, New Lambton Heights, NSW, Australia; ^7^MRC Laboratory of Molecular Biology, Francis Crick Avenue, Cambridge Biomedical Campus, Cambridge, United Kingdom; ^8^Centre for inflammation, Centenary Institute, Sydney, NSW, Australia; ^9^Faculty of Science, University of Technology, Ultimo, NSW, Australia

**Keywords:** ILC2, group 2 innate lymphoid cell, IL-5, IL-13, kidney, renal, IRI, ischemia-reperfusion injury

## Abstract

Acute kidney injury (AKI) can be fatal and is a well-defined risk factor for the development of chronic kidney disease. Group 2 innate lymphoid cells (ILC2s) are innate producers of type-2 cytokines and are critical regulators of homeostasis in peripheral organs. However, our knowledge of their function in the kidney is relatively limited. Recent evidence suggests that increasing ILC2 numbers by systemic administration of recombinant interleukin (IL)-25 or IL-33 protects against renal injury. Whilst ILC2s can be induced to protect against ischemic- or chemical-induced AKI, the impact of ILC2 deficiency or depletion on the severity of renal injury is unknown. Firstly, the phenotype and location of ILC2s in the kidney was assessed under homeostatic conditions. Kidney ILC2s constitutively expressed high levels of IL-5 and were located in close proximity to the renal vasculature. To test the functional role of ILC2s in the kidney, an experimental model of renal ischemia-reperfusion injury (IRI) was used and the severity of injury was assessed in wild-type, ILC2-reduced, ILC2-deficient, and ILC2-depleted mice. Surprisingly, there were no differences in histopathology, collagen deposition or mRNA expression of injury-associated (*Lcn2*), inflammatory (*Cxcl1, Cxcl2*, and *Tnf*) or extracellular matrix (*Col1a1, Fn1*) factors following IRI in the absence of ILC2s. These data suggest the absence of ILC2s does not alter the severity of renal injury, suggesting possible redundancy. Therefore, other mechanisms of type 2-mediated immune cell activation likely compensate in the absence of ILC2s. Hence, a loss of ILC2s is unlikely to increase susceptibility to, or severity of AKI.

## Introduction

Acute kidney injury (AKI) and its associated pathologies have profound effects on human health (1). A common cause of AKI is renal ischemia, which primarily affects the tubular epithelium due to the high mitochondrial density and metabolic activity in these cells (2); reviewed in Devarajan (3) and Bonventre and Yang (4). Acute tubular necrosis impairs waste excretion, alters water and electrolyte imbalance, and causes robust inflammatory responses; reviewed in Bonventre and Yang (4). Influx of neutrophils and monocytes contributes to the injury, however other innate immune cells can facilitate the return to homeostasis (5); reviewed in Friedewald and Rabb (6). Innate lymphoid cells (ILCs) are a recent addition to this family and are categorized into 3 groups based on the transcription factors that are required for their development and by the effector cytokines they produce (7). Group 2 ILCs (ILC2s) are potent producers of type 2 cytokines, predominantly interleukin (IL)-5 and IL-13 (8, 9). These cells also promote tissue recovery following insult in multiple organs and have diverse functions *in vivo* (10). For example, following respiratory viral infection with influenza virus, ILC2s co-ordinate repair of the airway epithelium by producing the growth factor amphiregulin (AREG) (11). More recently, ILC2s have been investigated in models of renal injury including ischemia-reperfusion injury (IRI), and nephrotoxic chemical-induced injury with doxorubicin or cisplatin (12–16). Renal IRI is typically achieved by temporarily restricting blood flow to the kidney for 20–30 min, modeling trauma from transplant or surgical intervention (17–21). Collectively, these studies show that *in vivo* administration of recombinant mouse cytokines that activate ILC2s, namely IL-25 or IL-33, is sufficient to reduce the severity of tubular epithelial cell injury (12–16). Similar results have been achieved with adoptive transfer of *ex vivo* activated ILC2s (12, 15). Whilst ILC2s can be artificially induced to proliferate and protect against the deleterious consequences of experimental renal injury, the impact of ILC2-deficiency remains incompletely understood. Indeed, other immune cells, which have complex interactions with the ILC2s such as regulatory T cells (T_reg_) and alternatively activated macrophages (AAM; also known as M2) are critical for this renoprotective effect (22–25). In this study, we sought to further characterize ILC2s in the kidney, their location within this organ and determine their functional role in IRI using a loss-of-function approach. Here, we found that kidney ILC2s constitutively express IL-5 and are primarily located in close proximity to the renal vasculature, within the adventitia. Additionally, we demonstrate that a reduction, deficiency or depletion of ILC2s had minimal impact on the severity of IRI. Whilst activation of ILC2s and the associated amplification of local type 2 immunity has been previously shown to reduce the deleterious consequences of AKI, our results reveal that comparable injury occurs in the absence of ILC2s, suggesting that ILC2s may be redundant in IRI when other compensatory immune cells such as T cells are present.

## Materials and Methods

### Mice

Eight to twelve week-old male wild-type (WT; C57BL/6JAusb), vehicle (saline-treated *Icos*^dtr/+^*Cd4*^cre/+^), ILC2-reduced (*Rora*^fl/+^*Il7r*^cre/+^), ILC2-deficient (*Rora*^fl/fl^*Il7r*^cre/+^), ILC2-depleted (Diptheria toxoid-treated [DTx] *Icos*_dtr/+_ *Cd4*_cre/+_), and IL-5/IL-13 dual reporter mice (*Il5*^venus/+^*Il13*^td−tomato/+^) mice were obtained from Australian Bioresources (Moss Vale, Australia). *Il5*^td−tomatoCre^; Rosa26-CAG-RFP reporter-tracker mice were obtained from Jackson Labs (ref# 030926 and 007914). All mice that underwent surgery were housed under specific pathogen free, physical containment 2 conditions, in individually ventilated cages. Mice were exposed to normal room air within a sterilized environment during surgery. Mice were allowed 1 week to acclimatize before experiments were started and were maintained on a 12-h day/night cycle with access to standard laboratory chow and water *ad libitum*.

### Flow Cytometry and t-SNE Analysis

Kidneys and lungs were collected from reporter mice. Single-cell suspensions were prepared as described in “Preparation of single-cell suspensions from mouse lung with Collagenase D treatment” (Miltenyi Biotec GmbH, 2008; Bergisch Gladbach, Germany). Cells were blocked with Fc block (purified anti-mouse CD16/32; Biolegend, San Diego, USA) for 30 min and stained with fluorescently-conjugated antibodies against target cell surface antigens (Supplementary Table 1). Staining and washing steps were performed with BSA stain buffer (BD Biosciences, North Ryde, Australia). Samples were acquired on a BD LSR Fortessa X20 flow cytometer. Flow cytometry data were analyzed using FlowJo v10.5.3 (Tree Star, Ashland, USA). The t-distributed stochastic neighbor embedding (t-SNE) was performed on ILC2s (CD45^+^Lineage^−^[TCR^−^[TCRαβ^−^TCRγδ^−^CD8^−^CD4^−^]CD11b^−^GR-1^−^B220^−^TER-119^−^CD3^−^NK-1.1^−^]IL7Rα^+^CD90.2^+^ST2^+^ FSC^low^SSC^low^ single cells). Using the random down sampling plugin, the number of events in each sample was normalized to equal the lowest, rounded down to the nearest 10 events. All populations were combined into one .fcs file by concatenating the down sampled populations. The t-SNE analysis was performed on the concatenated sample containing the gated populations from all kidney and lung samples combined. For this, the compensated channels that were not used for gating were assessed under the following t-SNE settings: Iteration 1000, Perplexity 20, Eta 224. Histograms were used to show the differential expression of cell surface antigens and cytokines in the lung and kidney.

### Immunofluorescence

*Il5*^td−tomatoCre^; Rosa26-CAG-RFP reporter-tracker mice were euthanized and perfused intracardially with phosphate buffered saline (PBS) and then 4% paraformaldehyde (PFA) in PBS. Kidneys were removed and kept in fresh 4% PFA for 24 h at 4°C and then washed in PBS. Three hundred micrometer sections were prepared using a vibratome. Subsequently, tissue sections were incubated in permeabilization buffer (PBS/0.2% Triton X-100/0.3M glycine) then blocked in PBS/0.2% Triton X-100/5% donkey serum at 4°C overnight. Samples were washed in PBS/0.2% Tween-20 once, then incubated with primary antibodies diluted in PBS/0.2% Tween-20/3% donkey serum at room temperature until the next day. Next, samples were washed in PBS/0.2% Tween-20 for 30 min, 3–4 times, then incubated with secondary antibodies diluted in PBS/0.2% Tween-20/3% donkey serum at room temperature for 6–8 h. Samples were washed in PBS/0.2% Tween-20 for 1 day and then dehydrated in an ascending ethanol series (20, 30, 50, 70, 95, 100%), 10 min each step. Finally, kidney sections were cleared by soaking in methyl salicylate (M-2047; Sigma-Aldrich, Castle Hill, Australia) and then mounted in fresh methyl salicylate onto a concave coverslip or chamber. Images were captured with Nikon A1R laser scanning confocal including 405, 488, 561, and 650 laser lines for excitation and imaging with 16X/0.8 or 25X1.1, NA Plan Apo long working distance water immersion objectives. Z steps were acquired every 6 μm. Images were processed with ImageJ (NIH, Bethesda, MD, US) and Bitplane Imaris software v8 (Andor Technology PLC, Belfast, N. Ireland). Surface reconstructions of alpha smooth muscle actin-labeled blood vessels were performed with Imaris and pseudocolored based on their characteristic architecture and volume to visualize large arteries and veins. The following antibodies and dilutions were used: Living Colors anti-DsRed Rabbit Polyclonal Pan Antibody (1:500; TaKaRa, Mountain View, USA), goat polyclonal anti-alpha smooth muscle actin antibody (1:200; Abcam, Cambridge, UK), eFluor 660 LYVE1 monoclonal Antibody (1:300, clone ALY7, eBioscience), armenian hamster anti-mouse CD3 antibody (1:100, clone 145-2C11, Biolegend), Alexa Fluor® 488 donkey anti-goat IgG (H+L), Alexa Fluor® 555 donkey anti-rabbit IgG (H+L) Alexa Fluor® 647 goat anti-hamster IgG (H+L) cross-adsorbed (1:400, ThermoFisher Scientific).

### Unilateral Ischemia-Reperfusion Injury

Surgical anesthesia was induced by 4% isoflurane inhalation and maintained with continuous 1–1.5% isoflurane inhalation through a nose cone. Throughout the procedure, mice were kept on a surgical platform maintained at 37 ± 1°C by a digital thermostat-controlled heat mat. Nephrectomy of the right kidney was performed in all animals. To induce unilateral ischemia the vessels of the left kidney were clamped near the renal pedicle for 29-min using a non-traumatic sterile vascular clamp (Coherent Scientific; Hilton, Australia) applying sufficient force to prevent blood flow to and from the kidney without irreversible damage to the vessels. Uniform and rapid reperfusion of the kidney was observed after removing the vascular clamp in all mice. The muscle and skin wounds were closed using a 5/0 vicryl suture (Johnson & Johnson Medical, North Ryde, Australia), the skin was then sealed using dermal adhesive (Provet, Charlestown, Australia) to protect the wound and prevent infection. Control animals were subjected to a sham operation with identical anesthesia time and received only a contralateral nephrectomy. For effective analgesia mice received 0.1 mg/kg of buprenorphine subcutaneously 30 min prior to the operation and then 0.05–0.1 mg/kg every 8–12 h, as required. Animals were typically weaned off analgesia by 4 days post-surgery without any signs of pain or distress as defined by facial characteristics using the standard mouse grimace scale; as described (26). All mice survived to the pre-determined endpoint.

### Depletion of ILC2s *in vivo*

ILC2s were depleted in *Icos*^dtr/+^*Cd4*^cre/+^ mice by intraperitoneal injection of 0.025 mg/kg of DTx (Sigma-Aldrich) as previously described (27). DTx was administered 30 min prior to surgery and daily thereafter until the endpoint. To confirm ILC2-depletion, a subset of naïve animals received DTx for 3 days. Control *Icos*^dtr/+^*Cd4*^cre/+^ mice received intraperitoneal saline injection.

### Histology

Excised kidneys were immediately sectioned in half longitudinally through the hilum. Half was fixed in 10% buffered formalin, containing 4% formaldehyde for 24 h before temporary storage in 10% ethanol, paraffin embedding, and sectioning. Four micrometer, longitudinally cut sections were stained with periodic acid-Schiff (PAS) and Masson's trichrome. Histopathology was quantified by visualizing dilation of tubules, apoptosis or cast formation by a single blinded investigator using a well-established semi-quantitative method (28), with minor alterations as described (15). An average was taken of the score from duplicate PAS stained sections from each animal. A score of 0 = 0% of tubules were affected by the above histopathological features; 1 = 1–10%; 2 = 11–25%; 3 = 26–50%; 4 = 51–75%; 5 = 76–100%. Collagen deposition was assessed using Masson's trichrome and collagen was identified as methyl blue content between adjacent tubules.

### RNA Extraction, Reverse Transcription, and qPCR

Half of one kidney was retained for gene expression analysis. The tissue was homogenized in 400 μl of PBS using a TissueLyser LT™ (Qiagen, Chadstone Centre, Australia) through 50 oscillations per second for 2 min. Two hundred microliter of the sample was removed for other analyses. One milliliter of TRI Reagent® (Sigma-Aldrich) was added to the remaining 200 μl of the sample, which received a further 50 oscillations per second for 1–2 min until completely homogenized. Following 5 min of incubation at room temperature and centrifugation at 12,000x *g* for 10 min, the supernatant was extracted and vortexed with 250 μl of chloroform (Sigma-Aldrich). After incubating for 10 min at room temperature and centrifugation at 12,000x *g* for 15 min, the supernatant was extracted and vortexed with 500 μl of 70% isopropanol (Sigma-Aldrich). The sample was incubated again for 10 min at room temperature then was centrifuged at 12,000x *g* for 10 min. The supernatant was discarded without disrupting the RNA-rich pellet. The pellet was washed twice with 1 ml of 75% RNA-grade ethanol (Sigma-Aldrich), centrifuged at 10,000x *g* for 5 min and the supernatant was discarded each time. The pellet was air-dried at room-temperature briefly before re-suspending in 80–100 μl of UltraPure™ DNase/RNase-free double distilled water (ddH_2_O; Thermo-Fisher Scientific) and was then stored at −80°C prior to reverse transcription. Immediately prior to reverse transcription, RNA yield was determined by spectrophotometric assessment using a Nanodrop2000™ (Thermo-Fisher Scientific). Five microliter of each sample was diluted in ddH_2_O such that 1,000 ng of RNA was loaded per sample. Following DNAse (Sigma-Aldrich) treatment, cDNA was generated using the M-MLV (Thermo-Fisher Scientific, North Ryde, Australia) enzyme standard operating procedures using a T100™ thermal cycler (BioRad Laboratories, Gladesville, Australia). Using SYBR based methodology, qPCR was performed using CFX384 Touch™ (BioRad Laboratories) as per standard operating procedures. Each primer set was gradient tested to determine the optimal temperature for amplification (Supplementary Table 2). Cycle quantification was interpreted using regression for each target, gene expression was normalized relative to hypoxanthine guanine phosphoribosyl transferase (*Hprt*). Melt curves were assessed for signs of primer dimerization and non-specific amplification. On every plate, at least one negative control and no-template control was used for each target to ensure contamination was not present within each master mix or the ddH_2_O used.

### RT^2^ Profiler Array

RT^2^ Profiler™ PCR Array Mouse Extracellular Matrix & Adhesion Molecules (Qiagen) was performed as per manufacturer's instructions and data was uploaded then analyzed by the online tool, available from: https://dataanalysis.qiagen.com/pcr/arrayanalysis.php. This array allows profiling of 84 genes in a maximum of 4 samples, therefore, equal amounts of cDNA for each replicate in a group were pooled from WT mice on day 1, 3, or 7 following IRI, and sham pooled from each replicate from each of these timepoints. For each target, the fold change following IRI was compared to sham expression.

### Statistical Analysis

All data were analyzed with Graphpad Prism software v8.02 using non-parametric unpaired *t*-tests (Mann-Whitney *U*-test). *P* < 0.05 was set as threshold for determining statistically significant differences. ^*^P < 0.05, ^ns^ not significant. All data are expressed as mean ± SEM. In each analysis there were *n* = 4–8 replicates per group and results were representative of at least two experiments. Sample size for each experiment is described in the corresponding figure legend.

## Results

### Renal ILC2s Constitutively Express IL-5 and Are Localized to the Renal Vasculature Under Homeostatic Conditions

**Figure 1:**
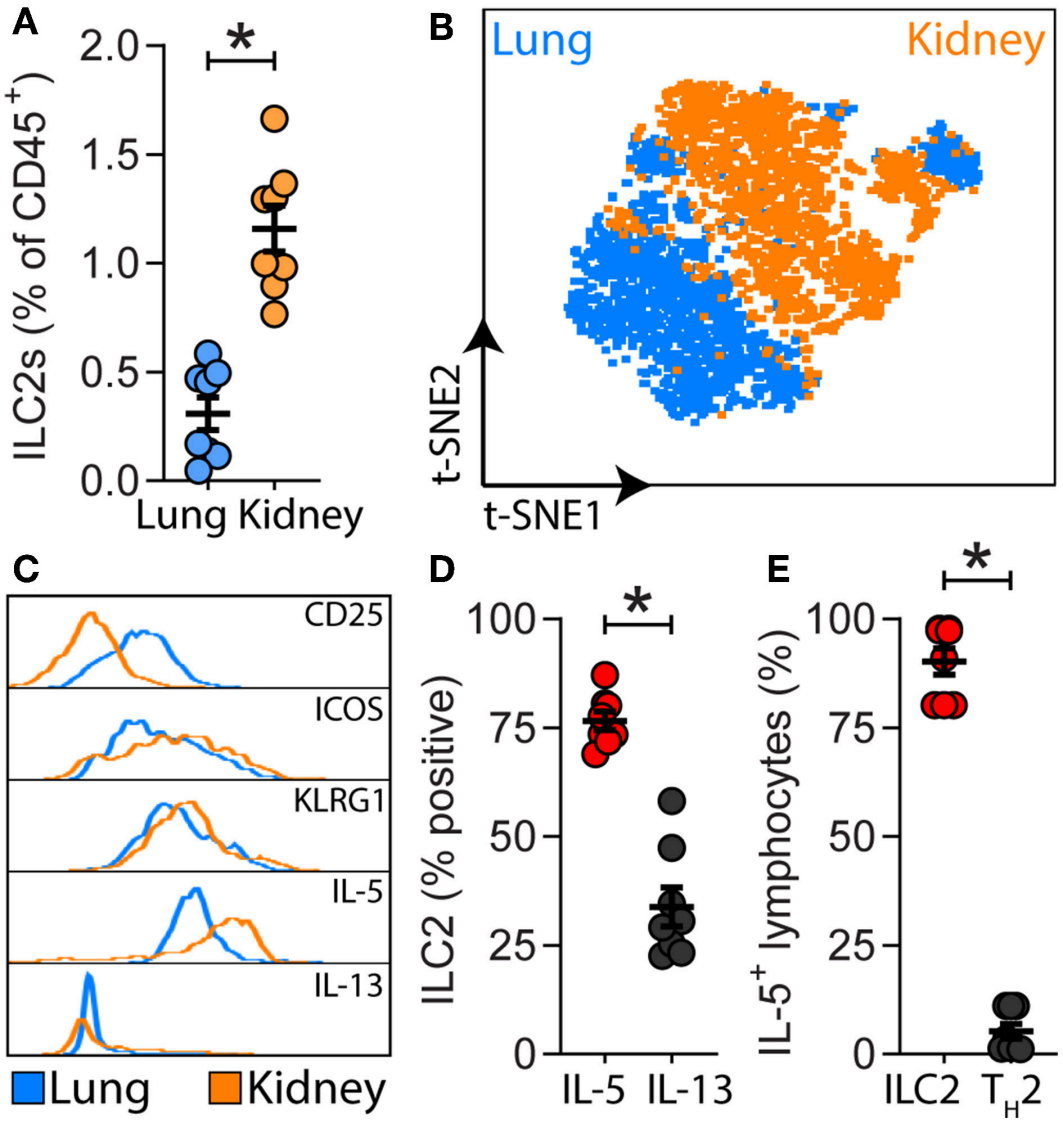
ILC2s are present in the kidney and have a unique signature compared to lung ILC2s. **(A)** ILC2s (CD45^+^Lineage^−^[TCR^−^[TCRαβ^−^TCRγδ^−^CD8^−^CD4^−^]CD11b^−^GR-1^−^B220^−^TER-119^−^CD3^−^NK-1.1^−^]IL-7Rα^+^CD90.2^+^ST2^+^FSC^low^SSC^low^ single cells) in kidney and lung single cell suspensions from naïve *Il5*^venus/+^*Il13*^td−tomato/+^ mice, *n* = 8. **(B)** Comparison of lung and kidney ILC2s by t-distributed stochastic neighbor embedding (t-SNE) analysis based on the cell surface antigens CD25, ICOS, KLRG1, and the type 2 effector cytokines IL-5 and IL-13. **(C)** Differential expression of ILC2-associated cell surface antigens and type 2 effector cytokines. **(D)** Percentage of IL-5^+^ and IL-13^+^ renal ILC2s. **(E)** Proportions of CD45^+^CD19^−^CD11b^−^CD49b^−^CD90.2^+^IL-5^+^ single cells which were consistent with ILC2s (CD3^−^CD4^−^) and T_H_2s (CD3^+^CD4^+^) in kidney single cell suspensions from *Il5*^td−tomatoCre^; Rosa-CAG-RFP mice, *n* = 8. All data are expressed as mean ±SEM. ^*^P < 0.05 by Mann-Whitney *U*-test.

**Figure 2:**
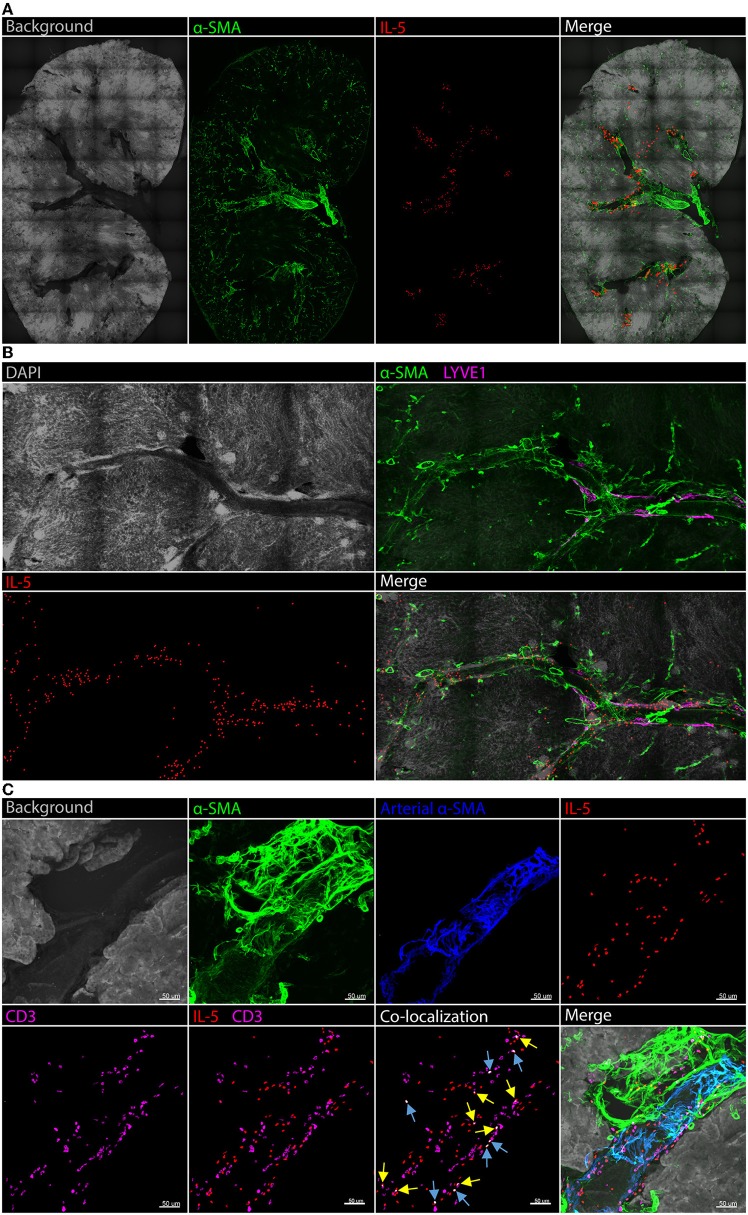
Kidney ILC2s are localized around the vasculature under homeostatic conditions. Kidney sections from *Il5*^td−tomatoCre^; Rosa-CAG-RFP mice were stained for IL-5^+^ cells (predominantly ILC2s), the IL-5^+^ pixel surface area was increased to improve clarity, tissue sections were co-stained for α-SMA for structure determination. **(A)** Gross structure of the kidney, ILC2s were located in close proximity to the renal vessels throughout the tissue. **(B)** A magnified view of a vessel spanning regions of the kidney with the addition of DAPI; LYVE1 staining indicates lymphatics which track vasculature in the cortex, ILC2s displayed no preference toward medullary or cortical vessels. **(C)** CD3^+^ cells (predominantly T_H_2 cells) contributed negligible IL-5^+^ signal. Background and arterial α-SMA staining demonstrates ILC2s and T_H_2 cells are located in the adventitia of the vessel. Blue arrows indicate IL-5 and CD3 co-localization in the same cell, yellow arrows indicate partial co-localization from adjacent cells.

A detailed analysis of renal ILC2 phenotype and location under homeostatic conditions was performed. There were significantly more ILC2s (CD45^+^Lineage^−^IL-7Rα^+^CD90.2^+^ST2^+^FSC^low^SSC^low^ single cells), in the kidney compared to the lung when represented as a percentage of total CD45^+^ single cells (Figure 1A; Supplementary Figures 1A, B). t-SNE analysis of kidney and lung ILC2s showed unique clustering, although these clusters were not entirely distinct from one another (Figure 1B). However, kidney ILC2s had markedly lower expression of CD25, consistent expression of ICOS and KLRG1, but higher expression of IL-5 compared to lung ILC2s (Figure 1C). Kidney ILC2s had higher constitutive expression of IL-5 than IL-13 (Figure 1D; Supplementary Figures 1C, D). ILC2s were determined to be the major IL-5 producing cell type in the kidney, with a negligible contribution from T helper type-2 (T_H_2) cells (Figure 1E, Supplementary Figures 1E, F). Therefore, IL-5 was used as a surrogate marker to identify the location of ILC2s within the mouse kidney, as has been described for multiple peripheral organs (29–31). This was achieved by utilizing mice that express an IL-5 linked cre-recombinase crossed to a Rosa-tdtomato lineage tracker (30, 31). IL-5^+^ cells (predominantly ILC2s) were found throughout the kidney, but were primarily localized to the major vasculature, as visualized with alpha smooth muscle actin (α-SMA) positive staining (Figure 2A; Supplementary Figures 2A, B), consistent with our recent report (29). ILC2s were found to be associated with both interlobular and arcuate renal vessels in the cortex and medullary regions, as shown by lymphatic endothelial hyaluronan receptor-1 (LYVE1) expression (Figure 2B). Further imaging demonstrated that very few of the CD3^+^ cells (predominantly T_H_2 cells) contributed to the endogenous IL-5^+^ signal (Figure 2C). ILC2s were identified in the adventitia of the vessel using pseudocolored surface reconstructions of the arterial α-SMA.

### Reduction, Deficiency, or Depletion of ILC2s Does Not Alter the Severity of Experimental Renal Ischemia-Reperfusion Injury in Mice

**Figure 3:**
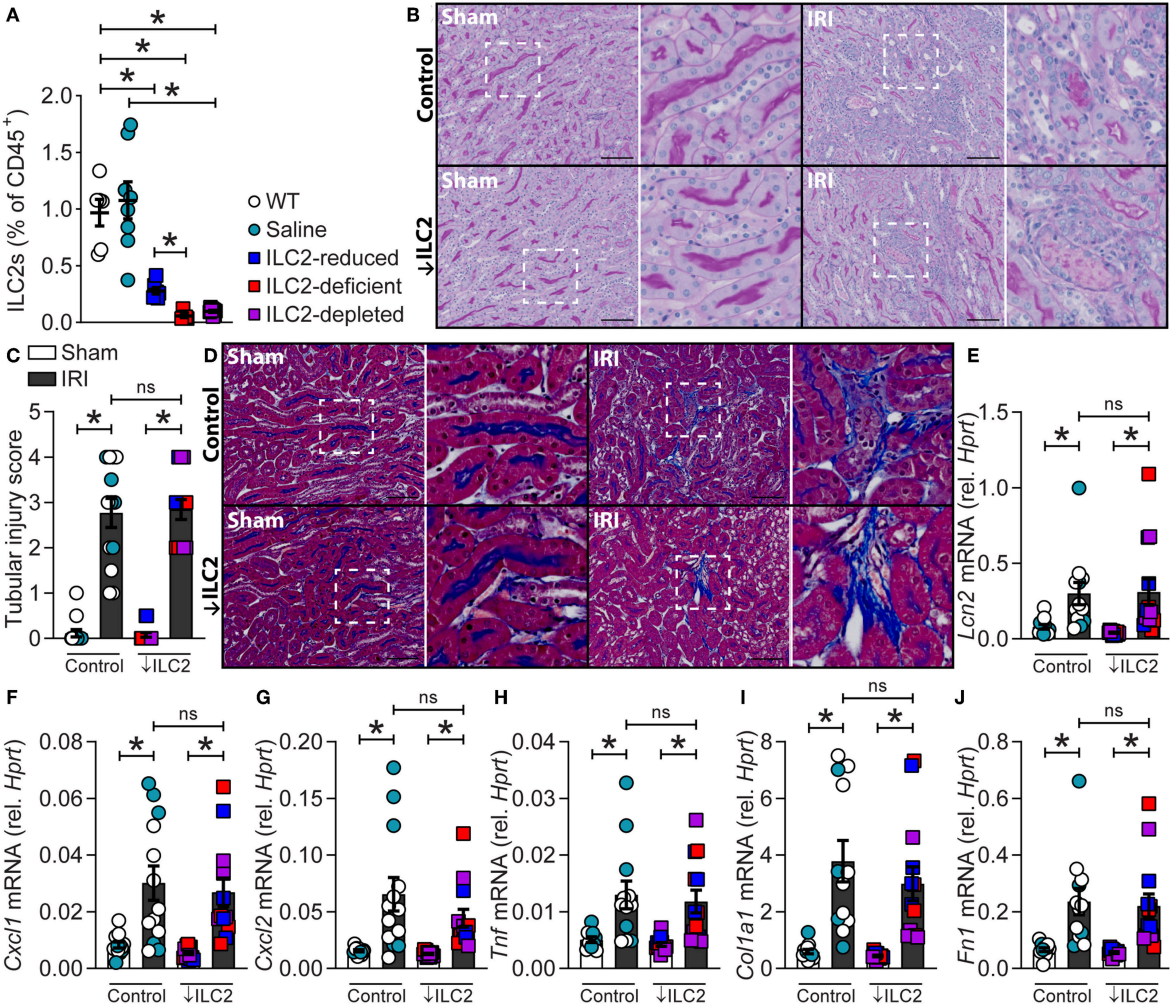
A reduction, absence or depletion of ILC2s does not alter the severity of experimental renal ischemia-reperfusion injury. All mice were subjected to 29-min unilateral IRI with contralateral nephrectomy and were assessed compared to sham surgical controls for each genotype. All parameters were assessed 7 days after injury. **(A)** Kidney ILC2s (CD45^+^Lineage^−^[TCR^−^[TCRαβ^−^TCRγδ^−^CD8^−^CD4^−^]CD11b^−^GR-1^−^B220^−^TER-119^−^CD3^−^NK-1.1^−^]IL-7Rα^+^CD90.2^+^ST2^+^FSC^low^SSC^low^ single cells) as a percentage of CD45^+^ single cells from naïve C57BL/6JAusB wild-type (WT, *n* = 6; uncolored), vehicle (saline-treated *Icos*^dtr/+^*Cd4*^cre/+^, *n* = 8; teal), ILC2-reduced (*Rora*^fl/+^*Il7r*^cre/+^, *n* = 7; blue), ILC2-deficient (*Rora*^fl/fl^*Il7r*^cre/+^, *n* = 5; red), and ILC2-depleted (DTx-treated-*Icos*^dtr/+^*Cd4*^cre/+^, *n* = 8; purple) mice. **(B)** Representative images of periodic acid-Schiff stained kidney sections from control (WT, *n* = 8; Saline, *n* = 5) and ↓ILC2 (ILC2-reduced, *n* = 4; -deficient, *n* = 4 & -depleted, *n* = 5) showing dilated tubules and cast formation. **(C)** Semi-quantitative tubular injury score indicating injury in terms of the proportion of tubules effected by casts, dilation, apoptosis, and/or loss of brush border, where a score of 5 indicates 76-100% of tubules were affected. **(D)** Representative images of Masson's trichrome stained kidney sections following IRI, blue staining indicates collagen deposition. **(E–J)** mRNA expression of injury (*Lcn2*), inflammatory (*Cxcl1, Cxcl2*, and *Tnf*), and extracellular matrix (*Col1a1* and *Fn1*) factors in kidney homogenates relative to *Hprt*. Scale bar in each image indicates 100 μm. All data are expressed as mean ± SEM. ^*^P < 0.05, ^ns^ not significant; by Mann-Whitney *U*-test.

Given ILC2s were present in the kidney and localized to the renal vasculature, the impact of ILC2 reduction, deficiency or depletion on the severity of experimental renal IRI was assessed. This was achieved by using ILC2-reduced (*Rora*^fl/+^*Il7r*^cre/+^), -deficient (*Rora*^fl/fl^*Il7r*^cre/+^), and -depleted (DTx-treated *Icos*^dtr/+^*Cd4*^cre/+^) mice compared to WT and vehicle (saline-treated *Icos*^dtr/+^*Cd4*^cre/+^) controls. These mice have been previously described as appropriate tools for assessing ILC2 function *in vivo* in other organs such as the lung (27, 32). Here we show that these tools are also appropriate for assessing ILC2 function in the kidney. Indeed, kidney ILC2s were significantly lower in the ILC2-reduced, -deficient, and -depleted groups compared to WT or saline-treated controls (Figure 3A). Time course analysis of IRI in wild-type mice identified day 7 as the most appropriate time point for the assessment of injury severity due to the presence of histopathological features of acute tubular necrosis, collagen deposition and increased mRNA expression of extracellular matrix, injury-associated, and inflammatory factors (Supplementary Figures 3, 4). At day 7, 25/84 extracellular matrix factors were increased >2-fold compared to sham controls, whilst 1/84 and 6/84 were increased at day 1 and 3, respectively (Supplementary Figure 4A). IRI induced acute tubular necrosis characterized by dilated tubules and cast formation, compared to sham controls (Figure 3B, Supplementary Figures 3A, 4B). This was quantified as a tubular injury score and the severity of injury was similar in all genotypes (Figure 3C). IRI also induced collagen deposition, marked by methyl blue content between the tubules in the medulla and cortex, compared to sham controls (Figure 3D; Supplementary Figure 3B). Collagen deposition was unaffected by the absence of ILC2s. IRI also increased the mRNA expression of injury-associated factors, chemokines, and pro-inflammatory cytokines (Figures 3E–J, Supplementary Figures 4C–K). Neutrophil gelatinase-associated lipocalin, (*Lcn2*; NGAL), a biomarker of renal injury (33, 34), was increased by IRI and unaffected by ILC2 reduction, deficiency, or depletion (Figure 3E). C-X-C motif chemokine ligand 1 (*Cxcl1*) and *Cxcl2* were also significantly increased by IRI in all genotypes (Figures 3F,G). Similarly, the pro-inflammatory cytokine tumor necrosis factor (*Tnf*) was increased by IRI, compared to sham controls (Figure 3H). Consistent with visible collagen deposition, mRNA expression of the extracellular matrix factors collagen type I alpha 1 chain (*Col1a1*), and fibronectin 1 (*Fn1*) were increased by IRI in all genotypes (Figures 3I,J).

## Discussion

ILC2s are critical regulators of tissue homeostasis, but their role in the kidney remains to be fully elucidated. Whilst ILC2s can be induced to proliferate and protect against the deleterious consequences of experimental renal injury, it is not yet known what occurs in the absence of these cells. We first examined whether ILC2 numbers, as a proportion of CD45^+^ hematopoietic cells, are different in lung and kidney. ILC2s accounted for a greater proportion of CD45^+^ cells in the kidney than in the lung. t-SNE analysis grouped ILC2s from both sites differently, however the clusters were not entirely distinct between these tissues. There were differences in their cell surface antigen and type 2 cytokine expression, with kidney ILC2s expressing greater amounts of IL-5. Indeed, kidney ILC2s constitutively expressed high levels of IL-5 under homeostatic conditions. ILC2s were also the major source of IL-5 in the kidney, with negligible contribution from T_H_2 cells. Therefore, IL-5 was used as a surrogate marker to determine the location of these cells in the kidney. ILC2s were identified within the kidney and were localized almost exclusively along the renal vasculature. In these studies, an IL-5 linked cre-recombinase was used in conjunction with a flox-stop-flox sequence upstream of a CAG-RFP-WPRE- cassette in the constitutively expressed ROSA26 locus to locate the ILC2s. LYVE1, which is expressed by renal lymphatic endothelial cells, was used to further define the location of these cells within the mouse kidney (35). As expected, LYVE1 staining was embedded within the connective tissue of renal arteries of the cortex, but not within the medulla since no discernable lymphatic network exists in this region of the kidney (35–37). Presumably ILC2s are situated close to the vascular as renal endothelial cells are a major source of IL-33 in the kidney (38, 39). Confirming our observation by FACS of single cell kidney suspensions, ILC2s were again found to be the major producers of IL-5 in kidney tissue sections, with minimal contribution from CD3^+^ T cells. Whilst these studies show the location of ILC2s in the kidney using reporter mice, it remains to be determined if ILC2s are also located adjacent to the vasculature in the human kidney, and whether the location of ILC2s is altered in response to renal injury. Although multiple reports have found ILC2 numbers are unchanged by renal injury, their phenotype and activation state following injury requires further elucidation (12–16). Next, we characterized the functional role of ILC2s in the kidney using a loss-of-function approach. To achieve this, we took advantage of ILC2-reduced and -deficient mice, and mice that can be conditionally depleted of ILC2s following the administration of DTx (27). We validated the use of these tools for knockdown or ablation of ILC2s in the kidney. An experimental model of IRI was chosen, given the proximity of ILC2s to the renal vasculature. Although features of injury were visibly evident following IRI, a reduction, deficiency, or depletion in ILC2s did not alter gross histopathology in the kidney, nor did it cause mortality. Indeed, IRI-induced remodeling and collagen deposition occurred independent of ILC2s. Whilst it is possible that an earlier time point following injury may have identified differences in histopathology and gene expression, the time point in our study also allowed assessment of collagen deposition, an important feature of remodeling that is regulated by ILC2s (16, 32, 40–44). It is also plausible that a reduction in ILC2s during AKI could be detrimental to kidney structure and function, when assessed at later time points due to potential maladaptive repair responses in the absence of ILC2s. To address this, future studies will need to investigate the role of ILC2s in the progression of AKI to chronic kidney disease (45, 46).

Collectively, this study demonstrates that a reduction, deficiency or depletion in ILC2s does not alter the severity of experimental AKI in mice. Whilst activation of ILC2s and the associated amplification of local type 2 immunity has been previously shown to reduce the deleterious consequences of AKI, comparable injury occurs when ILC2s are reduced, absent or depleted suggesting possible redundancy and compensation by other immune cells, such as T_reg_, AAM, and T_H_2 cells. This concept is supported by studies using *Rag1*^−/−^ or *Rag2*^−/−^ mice that lack mature T and B cells, in models of kidney injury (13, 15, 47, 48). In one study, anti-CD90.2 administration was used to deplete ILC2s, however these animals were also deficient in T_reg_ and T_H_2 cells (15). In this model system, despite the depletion of ILC2s with anti-CD90.2, compensation from the T cell compartment is not possible. Our data show that a loss of ILC2s, when the T cell compartment remains intact, has minimal effects on the severity of IRI. Our data supports the concept that the ILC2s in addition to other immune cells, such as T_reg_ and AAM, contribute to AKI. Indeed, T_reg_ depletion worsened histopathology following IRI (15). Similarly, macrophage polarization toward AAM promotes the resolution of injury (5, 12, 46, 49, 50). Further studies are required to elucidate the reason for the presence of ILC2s in the kidney, including ascending urinary tract infections and other renal insults as well as in progression to chronic disease.

## Ethics Statement

All surgical procedures were approved by The University of Newcastle's Animal Care and Ethics Committee in accordance with the Australian code for the care and use of animals for scientific purposes. Procedures for immunofluorescent imaging were approved and performed in accordance with guidelines established by UCSF Animal Care and Use Committee and Laboratory Animal Resource Center.

## Author Contributions

The work presented was performed in collaboration with all authors. GC performed the surgical model, flow cytometry, histological assessment, gene expression analysis, and analyzed the data. KC and ABM performed the immunofluorescence experiments. SL aided with animal monitoring and flow cytometry. SJ and AD provided clinical insight for study design and surgical technique. ANM created, supplied and advised on the use of ILC2-deficient & -depleted and *Il13*^td−tomato^ mice. PF supplied and advised on the use of *Il5*^venus^ reporter line. PF and PH advised on experimental design. MS designed, supervised, and facilitated all aspects of the studies. All authors participated in the interpretation of data, preparation and editing of manuscript for intellectual content.

### Conflict of Interest Statement

ANM has grant funding from GSK and AstraZeneca/MedImmune. The remaining authors declare that the research was conducted in the absence of any commercial or financial relationships that could be construed as a potential conflict of interest.
