# Supplementary material for: Group 2 Innate Lymphoid Cells Are Redundant in Experimental Renal Ischemia-Reperfusion Injury
Source: Front Immunol. 2019 Apr 16;10:826. doi: 10.3389/fimmu.2019.00826 (PMC6477147; doi:10.3389/fimmu.2019.00826)
Supplement: Supplementary file 1 [file Data_Sheet_1.PDF]

Supplementary Material

SUPPLEMENTARY FIGURES AND TABLES

Supplementary Figures

Supplementary Figure 1 | Flow cytometry gating strategy for ILC2s. **(A)** Lung and **(B)** kidney single cell suspensions from $\mathit{Il5}^{\text{venus/+}}\mathit{Il13}^{\text{td-tomato/+}}$ mice, n=8, were gated for ILC2s ($CD45^{+}Lineage^{-}$[TCR^{-}[$TCR\alpha\beta^{-}TCR\gamma\delta^{-}CD8^{-}CD4^{-}$]$CD11b^{-}GR\text{-}1^{-}B220^{-}TER\text{-}119^{-}CD3^{-}NK\text{-}1.1^{-}$]$IL\text{-}7R^{+}CD90.2^{+}ST2^{+}FSC^{low}SSC^{low}$ single cells). **(C)** Representative FACS plots of IL-5 and **(D)** IL-13 expression in kidney ILC2s. **(E)** Gating strategy demonstrating the proportion of $CD45^{+}CD19^{-}CD11b^{-}CD49b^{-}CD90.2^{+}IL\text{-}5^{+}$ single cells which were consistent with **(F)** ILC2s ($CD3^{-}CD4^{-}$) and T_H2s ($CD3^{+}CD4^{+}$) in kidney single cell suspensions from $\mathit{Il5}^{\text{td-tomatoCre}}$; Rosa-CAG-RFP mice, n=8.

Supplementary Figure 2 | Immunofluorescence imaging of $IL\text{-}5^{+}$ cells in the kidney. Kidney sections from $\mathit{Il5}^{\text{td-tomatoCre}}$; Rosa26-CAG-RFP mice were stained with DAPI, α-SMA and DsRed to visualize nuclei, smooth muscle actin and IL-5, respectively. **(A)** Visualization of broad regions of the mouse kidney, $IL\text{-}5^{+}$ staining was identified in the calyx, capsule, cortex and medulla. **(B)** $IL\text{-}5^{+}$ staining was localized to blood vessels in the kidney. DsRed identified $IL\text{-}5^{+}$ cells and imaris software identified maximum projection surfaces and enhanced the pixel area for ease of visualization.

Supplementary Figure 3 | Histopathology following IRI was unaltered by the reduction of ILC2s. C57BL/6JAusB wild-type (WT, n=8) mice were subjected to 29-minute unilateral IRI with contralateral nephrectomy and were assessed at 1, 3 and 7-days post-surgery. WT, vehicle (saline-treated $\mathit{Icos}^{\text{dtr/+}}\mathit{Cd4}^{\text{cre/+}}$, n=5), ILC2-reduced ($\mathit{Rora}^{\text{fl/+}}\mathit{Il7r}^{\text{cre/+}}$, n=4), ILC2-deficient ($\mathit{Rora}^{\text{fl/fl}}\mathit{Il7r}^{\text{cre/+}}$, n=4) and ILC2-depleted (diphtheria toxoid-treated $\mathit{Icos}^{\text{dtr/+}}\mathit{Cd4}^{\text{cre/+}}$, n=5) were assessed 7-days post-surgery. **(A)** Representative images of periodic acid-Schiff and **(B)** Masson's trichrome stained kidney sections following sham and IRI surgery, blue staining indicates collagen. Scale bar in each image indicates 100μm.

Supplementary Figure 4 | IRI increased mRNA expression and histopathological score. **(A)** Extracellular Matrix & Adhesion Molecules RT^2 Profiler PCR Array, truncated to 25/84 targets, which were increased >2 fold in kidney homogenates from C57BL/6JAusB wild-type (WT, n=8) on day 1, 3 or 7 following IRI (n=8 per timepoint), compared to the average expression across each timepoint following sham surgery (n=24). **(B)** Semi-quantitative tubular injury score indicating injury in terms of the proportion of tubules effected by casts, dilation, apoptosis and/or loss of brush border, where a score of 5 indicates 76-100% of tubules were affected. **(C-K)** mRNA expression of injury (*Lcn2*), inflammatory (*Cxcl1*, *Cxcl2* and *Tnf*), extracellular matrix (*Col1a1* and *Fn1*), ILC2- and M2-macrophage associated factors (*Areg*, *Arg1* and *Mrc1*) in kidney homogenates relative to *Hprt*. All data are expressed as mean ±SEM. * $P<0.05$, ^{ns} not significant; by Mann-Whitney U-test.

Supplementary Tables

Supplementary Table 1

**Panel 1: ILC2**					
**Antigen**	**Product code**	**Clone**	**Fluorophore**	**Dilution factor**	**Source**
CD278 (ICOS)	565886	C398.4A	BV421	100	BD Biosciences
TCR-αβ	563221	H57-597	BV510	300	BD Biosciences
TCR-γδ	563218	GL3	BV510	300	BD Biosciences
CD4	563106	RM4-5	BV510	300	BD Biosciences
CD8a	563068	53-6.7	BV510	300	BD Biosciences
KLRG1	740553	2F1	BV650	100	BD Biosciences
CD45	550994	30-F11	PerCP-Cy5.5	200	BD Biosciences
CD127 (IL-7Rα)	562419	SB/199	PE-CF594	100	BD Biosciences
CD25	552880	PC61	PE-Cy7	50	BD Biosciences
IL-33R (ST2)	17-9335-82	RMST2-2	APC	50	eBioscience
CD11b	557960	M1/70	AF700	100	BD Biosciences
LY-6G/C (GR-1)	557979	RB6-8C5	AF700	100	BD Biosciences
CD45R (B220)	557957	RA3-6B2	AF700	100	BD Biosciences
TER-119	560508	TER-119	AF700	100	BD Biosciences
CD3	561388	17A2	AF700	100	BD Biosciences
NK-1.1	560515	PK136	AF700	100	BD Biosciences

CD90.2	561641	53-2.1	APC-Cy7	200	BD Biosciences
IL-5	-	-	Venus (BB515)	-	Reporter mice
IL-13	-	-	Td-tomato (PE)	-	Reporter mice
**Panel 2: ILC2 and T_H2**					
**Antigen**	**Product code**	**Clone**	**Fluorophore**	**Dilution factor**	**Source**
CD45	564279	30-F11	BUV395	400	BD Biosciences
CD90.2	140318	53-2.1	BV605	400	Biolegend
CD3	100236	17A2	APC	100	Biolegend
CD4	100557	RM4-5	BV711	200	Biolegend
CD8a	100750	53-6.7	BV785	200	Biolegend
CD49b	108918	DX5	Pacific Blue	300	Biolegend
CD19	115520	6D5	PE-Cy7	200	Biolegend
CD11b	101259	M1/70	BV650	200	Biolegend
IL-5	-	-	Td-tomato (PE)	-	Reporter mice

Supplementary Table 1 | Flow cytometry antibodies for assessing group 2 innate lymphoid cells (ILC2s; Panel 1) alone, or in conjunction with T helper type 2 cells (T_H2; Panel 2).

Supplementary Table 2

**Gene ID**	**Forward primer (5`-3`)**	**Reverse primer (5`-3`)**
Areg	TTGCTGCTGGTCTTAGGCTC	TGGTCCCCAGAAAGCGATTC
Arg1	GGCAGAGGTCCAGAAGAATG	GTGAGCATCCACCCAAATG
Ccl20	AGACGCCTCTTCCTTCGAGAGC	TGCTTTGGATCAGCGCACACA

Ccl5	TGGCTCGGACACTCCCTG	GGGTTGGCACACACTTGGCGG
Col1a1	CTTCACCTACAGCACCCTTGTG	TGACTGTCTTGCCCCAAGTTC
Cxcl1	GCTGGGATTCACCTCAAGAA	CTTGGGGACACCTTTTAGCA
Cxcl10	CCAAGTGCTGCCGTCATTTTC	TCCCTATGGCCCTCATTCTCA
Cxcl2	TGCTGCTGGCCACCAACCAC	AGTGTGACGCCCCCAGGACC
Fn1	GCACTGCTGCTGATTCAAGTTC	AGTTGCTCCTGGCTGGTATG
Hprt	AGGCCAGACTTTGTTGGATTTGAA	CAACTTGCGCTCATCTTAGGCTTT
Lcn2	TACAATGTCACCTCCATCCTGG	CCACTTGCACATTGTAGCTCT
Mrc1	CATGGATGTTGATGGCTACTGG	CCATAGAAAGGAATCCACGCAGT
Nos2	AGCGAGGAGCAGGTGGAAGACT	CCATAGGAAAAGACTGCACCGAA
Tnf	TCTGTCTACTGAACTTCGGGGTGA	TTGTCTTTGAGATCCATGCCGTT

Supplementary Table 2 | qPCR primer sequences.
