# Supplementary figures and images for: Group 2 Innate Lymphoid Cells Are Redundant in Experimental Renal Ischemia-Reperfusion Injury
Source: Front Immunol. 2019 Apr 16;10:826. doi: 10.3389/fimmu.2019.00826 (PMC6477147; doi:10.3389/fimmu.2019.00826)

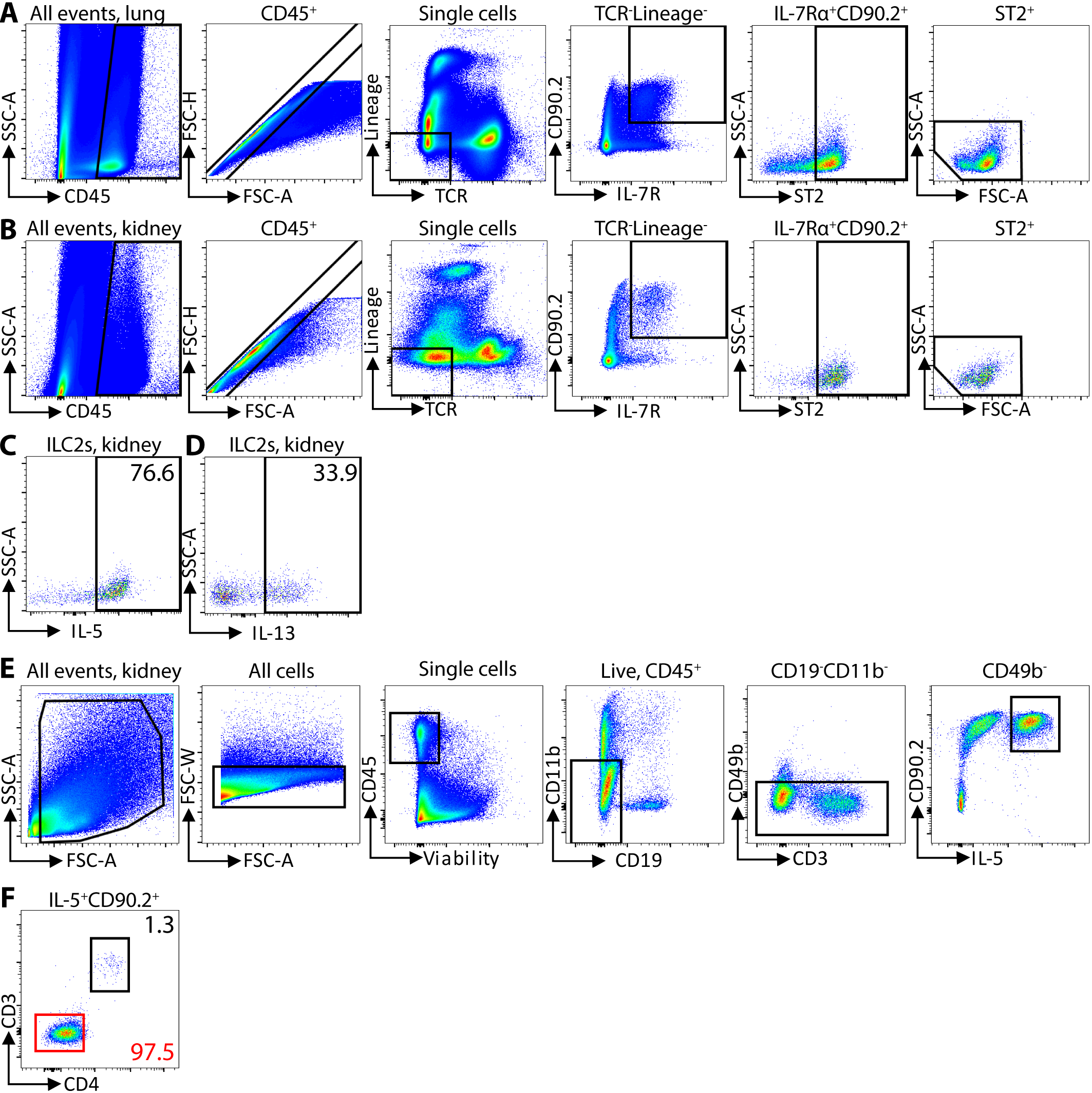

Supplement: Supplementary file 2 [file Image_1.TIF]

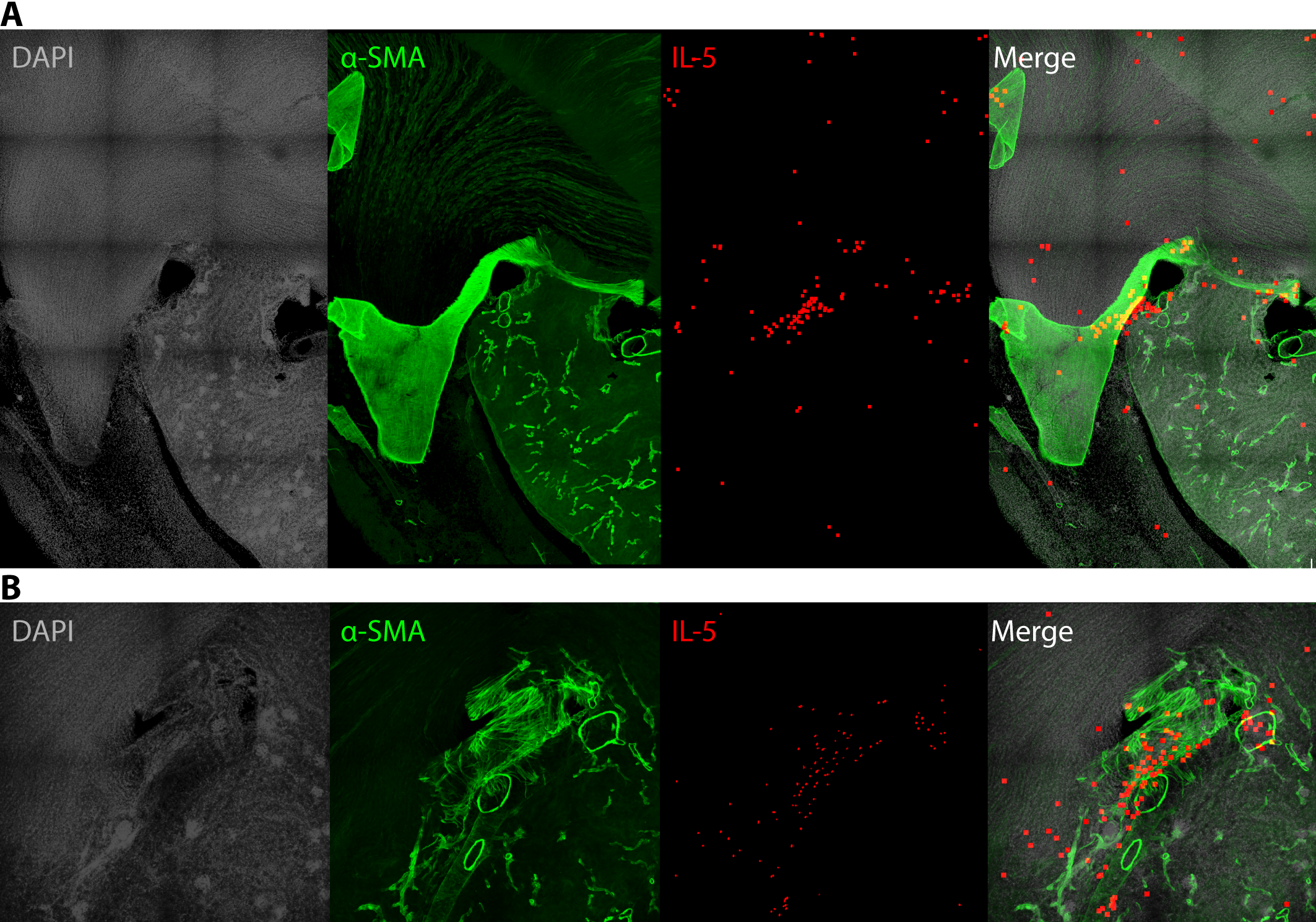

Supplement: Supplementary file 3 [file Image_2.TIF]

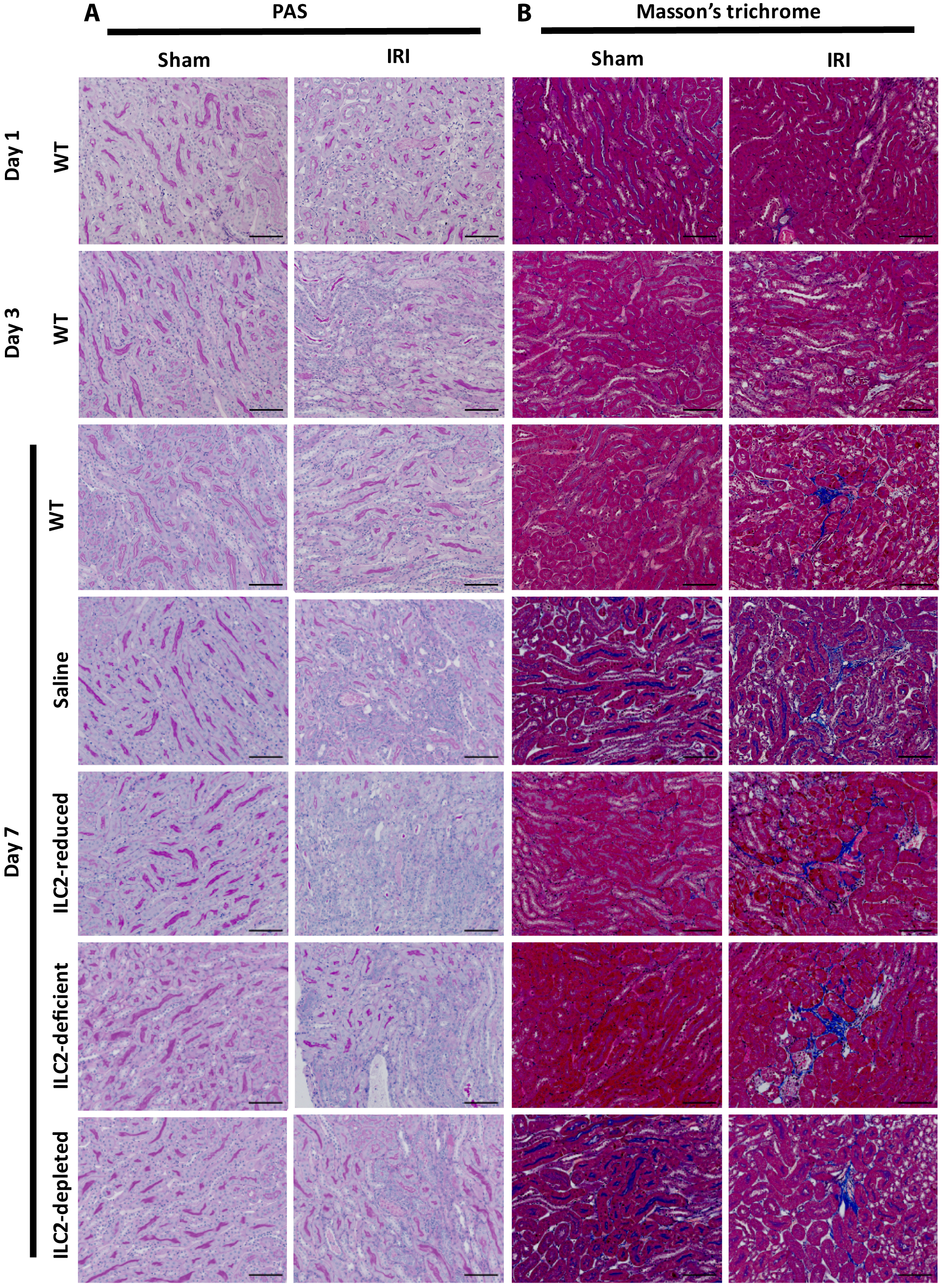

Supplement: Supplementary file 4 [file Image_3.TIF]

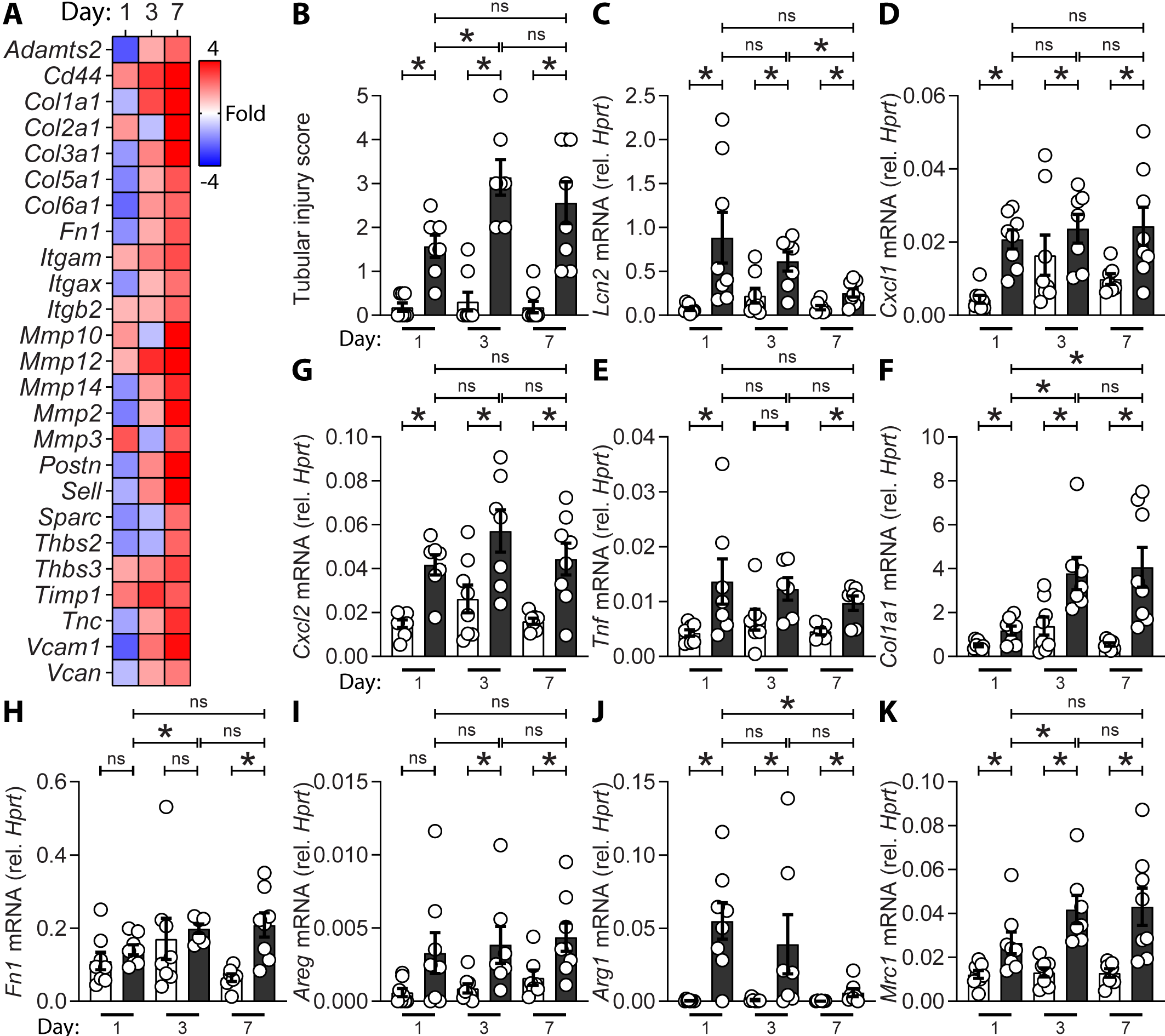

Supplement: Supplementary file 5 [file Image_4.TIF]
